# Allosteric coupling of the inner activation gate to the outer pore of a potassium channel

**DOI:** 10.1038/srep03025

**Published:** 2013-10-23

**Authors:** Christian J. Peters, David Fedida, Eric A. Accili

**Affiliations:** 1 Department of Cellular and Physiological Sciences, Vancouver, British Columbia, V6T 1Z3; 2 Department of Anesthesiology, Pharmacology, and Therapeutics, University of British Columbia, Vancouver, British Columbia, V6T 1Z3

## Abstract

In potassium channels, functional coupling of the inner and outer pore gates may result from energetic interactions between residues and conformational rearrangements that occur along a structural path between them. Here, we show that conservative mutations of a residue near the inner activation gate of the *Shaker* potassium channel (I470) modify the rate of C-type inactivation at the outer pore, pointing to this residue as part of a pathway that couples inner gate opening to changes in outer pore structure and reduction of ion flow. Because they remain equally sensitive to rises in extracellular potassium, altered inactivation rates of the mutant channels are not secondary to modified binding of potassium to the outer pore. Conservative mutations of I470 also influence the interaction of the *Shaker* N-terminus with the inner gate, which separately affects the outer pore.

Ion flow through voltage-gated (Kv) potassium channels is limited by at least two mechanisms taking place at opposite ends of the ion permeation pore. An inner gate is formed by the intracellular portion of the S6 helices of the four subunits coming together in a “bundle crossing”, which opens in response to membrane depolarizations to allow ions to flow^1^,^2^,^3^,^4^. Another gate is found at the outer end of the pore, which switches between a conducting and non-conducting conformation. Rearrangement of the outer gate, also known as the selectivity filter for its role in accommodating potassium and promoting its throughput over sodium, takes place following opening of the inner gate, and the two processes are functionally coupled; the closed conformation of the intracellular pore stabilizes the conducting conformation of the selectivity filter whereas an open inner conformation favors a modified filter structure that is accompanied by a decay of current, referred to as C-type inactivation^5^,^6^,^7^,^8^. Furthermore, inner gate opening is promoted by the non-conducting conformation of the selectivity filter^9^.

Energetic interactions between residues and associated conformational rearrangements along a structural path between the inner and outer pore may underlie their coupling^10^,^11^,^12^ and has been proposed to explain a set of high resolution crystal structures obtained from the potassium channel KcsA, from *Streptomyces lividans*^13^. KcsA is gated mainly by protons, rather than by voltage, but the proton-activated currents decay over time in a manner that is similar to C-type inactivation in Kv channels. A continuum of intermediate conformations of the aperture of the inner gate is correlated with reorientation and a decrease in potassium occupancy of the selectivity filter^13^,^14^. Also identified was a residue that lies between the inner pore lining helix and the backbone amino acids of the selectivity filter^15^. Snapshots of this residue, F103, show that it is rotated as the gate widens, and lies closer to the selectivity filter, which is concomitantly reoriented into a conformation with a lower capacity for potassium ions. One appealing interpretation of the correlated differences in structure and loss of potassium binding in the KcsA channel is that opening of the inner gate re-orients F103, which then physically interacts with the selectivity filter to modify it and limit conduction. Such an interpretation of the observed structures is supported by conservative mutations of F103 that predictably modify the decay of KcsA-mediated current. Cuello et al^15^ also provide two pieces of evidence that suggest a similar sequence of molecular events upon opening and role for a F103-equivalent residue, an isoleucine in *Shaker*-related channels. First, using the Kv1.2 crystal structure as a starting point, they show strong theoretical energetic interactions of I402 with a similar subset of residues in the selectivity filter (see Figure 1). Second, they show that alanine substitution of the F103-equivalent residue, I470, in the N-type inactivation-removed *Shaker* (*Shaker*-IR) reduces the rate at which current decays; this is consistent with previous studies showing that the I470C mutation in *Shaker*-IR can result in a strong reduction in current decay^16^,^17^.

Based on the aforementioned evidence, Cuello et al^13^,^15^ suggested that opening-induced perturbations in structure observed for KcsA promote inactivation in all Kv channels, but this provocative proposal remains uncertain for several reasons. First, it is not known if the array of solved structures of the KcsA channel identified is truly representative of different stages of the inactivation process or, even if it is, whether such a structural series of events is common among related channels^18^. Second, the slowed inactivation of *Shaker-IR* produced by alanine substitution of I470 may be secondary to altered coordination of potassium as this cation is known to bind to the outer pore and slow C-type inactivation^19^,^20^. For example, a mutation in the pore of *Shaker*, A463C, has been suggested to slow C-type inactivation by enhancing the occupancy of potassium in the outer pore^21^,^22^. Third, the events proposed by Cuello et al may not apply to the full-length *Shaker* channel, where ion flow is reduced rapidly and dramatically upon opening and the inner gate is impacted by its own association with the full N-terminus^23^,^24^. The N-terminus blocks current by binding within the inner pore cavity, which accelerates inactivation; this may be due to a reduction of potassium ions residing in the selectivity filter and to allosteric signalling from an inner gate that has difficulty in closing, both of which promote the non-conductive conformation of the selectivity filter^6^.

To test the hypothesis that the archetypal potassium channel, *Shaker*, undergoes C-type inactivation as proposed for *KcsA*, we combined mutagenesis, recording of ion flux by two microelectrode voltage clamp and tracking of conformational changes in the selectivity filter by monitoring a fluorescent reporter attached to an outer pore residue. By examining the impact of conservative mutations of I470 on slow inactivation and showing that the mutant channels remain sensitive to extracellular potassium, we provide critical support for a model in which this residue undergoes a re-orientation as the inner gate opens and widens, physically impinging on the selectivity filter to change its conformation and limit conduction, as has been suggested for F103 in the KcsA channel. We also suggest that I470 interacts functionally with the long N-terminus to control affinity of its interaction with the inner side of the channel and speed C-type inactivation.

## Results

### A set of conserved residues identifies a putative coupling pathway that is common among Shaker-related potassium channels

**Figure 1:**
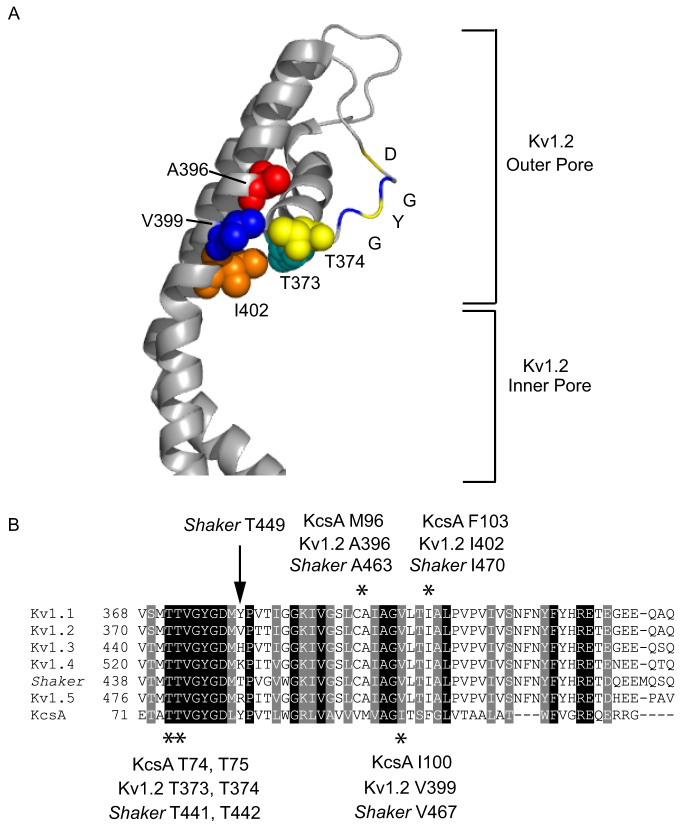
A set of conserved residues identifies a putative mechanism that is common among *Shaker*-related potassium channels. (A). The interface between the inner gate and pore helix of Kv1.2 (PDB #2R9R). Residues in Kv1.2 that are important for coupling of opening to rearrangement of the outer pore come from their equivalent positions in KcsA and are shown as Van der Waals spheres (equivalent KcsA/Kv1.2 positions); Met 96/Ala 396, red; Ile 100/Val 399, blue; Phe 103/Ile 402, orange; Thr 74/Thr 373, cyan; Thr 75/Thr 374, yellow. Also shown in color only, are four of the residues that form the selectivity filter; glycine (G, blue), tyrosine (Y,yellow), glycine (G,blue) and aspartate (D, yellow). (B). An alignment between *Shaker*, five human Kv1 isoforms and KcsA. Identical (black) and conserved (gray) residues among all of the isoforms are shaded. Stars beneath the alignment identify the residues in Kv1.2, *Shaker* and KcsA that correspond to the coupling pathway proposed by Cuello et al and include: Kv1.2 I402/*Shaker* I470, corresponding to phenyalanine 103 in KcsA, Kv1.2 V399/*Shaker* V467, corresponding to isoleucine 100 in KcsA, and Kv1.2 T373, T374/*Shaker* T441, T442, corresponding to threonine 74 and threonine 75 in KcsA. Also shown is an alanine, which corresponds to methionine 96 in KcsA; when mutated to cysteine in *Shaker*, the inactivation rate is modified and the affinity of the selectivity filter for potassium is reduced. The location of threonine 449 in *Shaker* is separately identified by an arrow in the alignment. The sequences shown are: human Kv1.1 (gi:119395748), human Kv1.2 (gi:4826782), human Kv1.3 (gi:88758565), human Kv1.4 (gi:4504817), human Kv1.5 (gi:25952087), *Shaker* (gi:288442), KcsA (gi:61226909). The alignment was carried out using Clustalw (http://bio.lundberg.gu.se/edu/msf2.html) and arranged into the figure by Boxshade 3.21 (http://www.ch.embnet.org/software/BOX_form.html).

Previously, calculations of interaction energies were carried out showing that a set of residues in Kv1.2 forms a pathway that couples the inner and outer pore gates as in KcsA (Figure 1)^15^. Isoleucine 402 (F103 in *KcsA*) was shown to make strong van der Waals contacts with threonine 373 and threonine 374 (T74, T75 in KcsA) in the P-helix portion of the outer pore. Additionally, phenylalanine 103 of KcsA makes a more subtle interaction with methionine 96, which corresponds to alanine 396 in Kv1.2, and a strong interaction with isoleucine 100, which corresponds to valine 399 in Kv1.2. The residues of Kv1.2 mentioned above are completely conserved with those of *Shaker* (Figure 1).

### Conservative substitutions at site I470 of Shaker modify C-type inactivation rate but maintain its sensitivity to extracellular potassium

**Figure 2:**
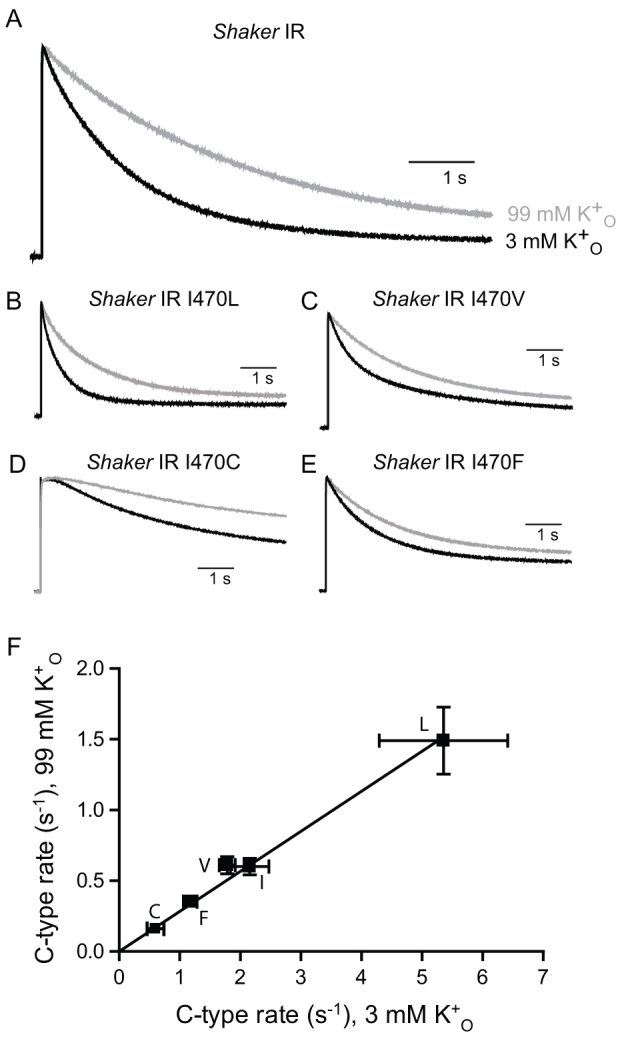
Extracellular potassium slows C-type inactivation in WT *Shaker* and I470 mutants. (A). Current traces from *Shaker*-IR in response to a voltage pulse to +60 mV from –80 mV, in solutions containing 3 mM (black) and 99 mM (grey) extracellular K^+^. Mean current decay rates (k) calculated from τ in 3 and 99 mM K^+^, respectively, were: 2.16 ± 0.32 s^−1^ and 0.60 ± 0.06 s^−1^, (n = 9) (B–E). Current traces using the same protocol from *Shaker*-IR I470L (B), I470V (C), I470C (D) and I470F (E). Current amplitudes in shown are normalized to the peaks of both traces. Mean current decay rates (k) calculated from τ's in 3 and 99 mM K^+^, respectively, were: for I470L, 5.35 ± 1.06 s^−1^ and 1.49 ± 0.24 s^−1^ (n = 6); for I470V, 1.78 ± 0.13 s^−1^ and 0.61 ± 0.06 s^−1^ (n = 8); for I470C, 0.62 ± 0.14 s^−1^ and 0.15 ± 0.01 s^−1^ (n = 4); for I470F, 1.17 ± 0.11 s^−1^ and 0.35 ± 0.03 s^−1^ (n = 8). I470V inactivation rates were calculated from the fast, K^+^-dependent value of τ. Time constant for I470C current inactivation was estimated by starting the fitting process after the “bump” at the beginning of the trace, which was apparent only in this mutant channel. (F). Inactivation rates from traces in A in 3 mM and 99 mM K^+^, determined using Equation 1, plotted against each other and fit by linear regression analysis (slope = 0.28 ± .01, R^2^ = 0.99).

To examine the coupling of the inner gate to the outer pore, we carried out mutagenesis of the I470 residue of the *Shaker* potassium channel in combination with electrophysiological approaches to quantify the rates of C-type inactivation. We began by studying the inactivation-removed *Shaker* channel, -IR, in which a portion of the distal N-terminus is removed; this eliminates a fast component of current decay and induced effects on C-type inactivation and the outer pore. Single conservative substitutions of I470 in *Shaker*-IR were introduced and the rates of current inactivation were determined. Inactivation was measured by pulsing oocytes expressing *Shaker*-IR from a holding potential of –80 mV to a test potential of +60 mV (Figure 2A); this test potential was chosen to maximally activate the channels and prevent any effect on current decay from the voltage-dependence of activation. That all of the mutant channels were not activated at −80 mV and maximally activated at +60 was confirmed from conductance-voltage (G-V) relationships that were determined for all constructs used in this study (data not shown).

When oocytes injected with constructs with conservative mutations of I470 to leucine (Figure 2B), valine (2C), cysteine (2D) and phenylalanine (2E) were given long depolarizing pulses, all showed altered rates of inactivation compared to the wild type construct (Figure 2A). *Shaker*-IR I470V inactivation traces required a double exponential function for adequate fitting. We treated the faster, K^+^-dependent time constant as the likely kinetic correlate of the process observed with the remaining constructs and used it for calculations and analysis. As found for KcsA^15^, smaller side chains generally showed greater impairment of inactivation.

The rate of C-type inactivation in Kv channels such as *Shaker* is sensitive to extracellular potassium, which occupies the outer pore and reduces this rate^19^,^20^. It is possible that the effects of the mutations to I470 on C-type inactivation are secondary to modified occupancy of potassium to the outer pore. To examine this possibility, we determined the rates of inactivation using an extracellular solution containing 99 mM potassium rather than 3 mM, in the same oocytes. With the higher concentration of extracellular potassium, the time course of inactivation was slowed in all I470 mutant constructs as compared to that made in the normal extracellular potassium concentration. When inactivation rates in normal and elevated extracellular potassium were plotted against each other, regression analysis demonstrated a linear relationship among the channels used indicating that the effect of raising extracellular potassium from 3 mM to 99 mM rates is preserved among them (Figure 2F).

The effects of I470 mutations can be compared and contrasted with those of A463C *Shaker-IR*, a mutation of alanine 463 in the pore (Figure 1) that also modifies (reduces) the rate of C-type inactivation^21^,^22^. However, unlike those for the I470 mutants, the tendency of C-type inactivation to be slowed by increasing the extracellular potassium concentration was not preserved for *Shaker-IR* A463C across a wide range of tested concentrations (Supplementary Figure 1), and in fact appeared to be slightly accelerated in the higher potassium concentration. The lack of sensitivity to extracellular potassium of this mutant is consistent with previous findings suggesting that slowed rate of C-type inactivation is due to elevated potassium occupancy of the outer pore, which we observed even with an extracellular solution containing a normal level of potassium, rather than to an effect on the conformational change that leads to C-type inactivation^21^,^22^.

### The relative effects of conservative substitutions of I470 in full-length Shaker are similar to those in Shaker-IR and remain sensitive to potassium

**Figure 3:**
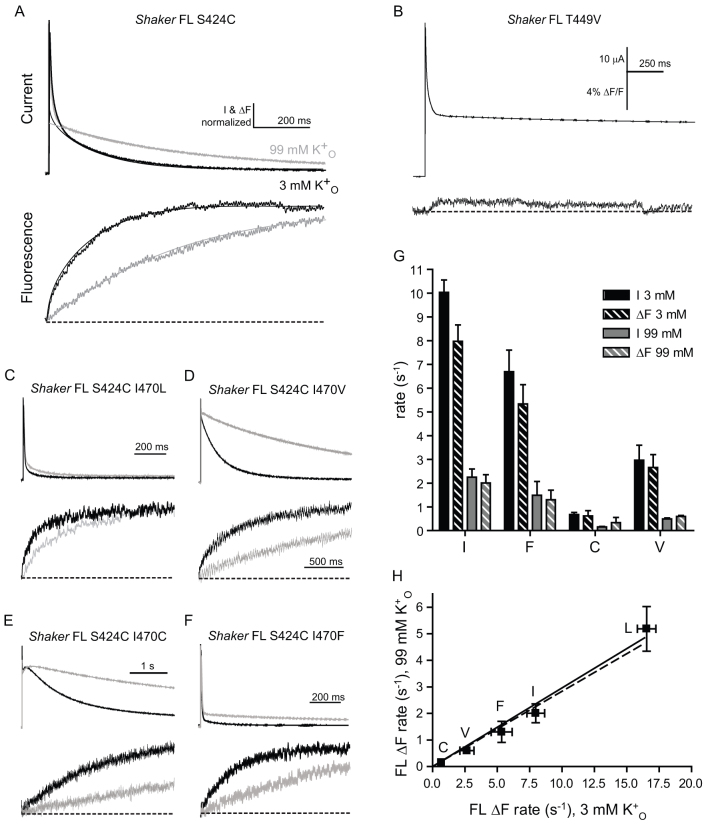
Extracellular potassium slows current decay equally in full-length *Shaker* and I470 mutants. (A). Current (top) and fluorescence (bottom) traces from *Shaker*-FL S424C in response to a voltage pulse to +60 mV from –80 mV, in solutions containing 3 mM (black) and 99 mM (grey) extracellular K^+^. Mean fluorescence decay rates (k) calculated from τ's in 3 and 99 mM K^+^, respectively, were: 7.98 ± 0.69 s^−1^ and 2.01 ± 0.35 s^−1^ (n = 5). Current and fluorescence amplitudes are normalized to one another for comparison. (B). Current and fluorescence traces from oocytes expressing *Shaker*-FL S424C T449V. (C–F). Current and fluorescence traces from the same protocol as in A applied to *Shaker*-FL S424C with the indicated amino acid substitutions. Mean fluorescence rates (k) calculated from τ's in 3 and 99 mM K^+^, respectively, were: (B) for I470L, 16.34 ± 0.71 s^−1^ and 5.19 ± 0.84 s^−1^ (n = 6); (C) for I470V, 2.66 ± 0.54 s^−1^ and 0.60 ± 0.04 s^−1^ (n = 5); (D) for I470C, 0.68 ± 0.09 s^−1^ and 0.15 ± 0.03 s^−1^ (n = 5); (E) for I470F, 5.34 ± 0.81 s^−1^ and 1.30 ± 0.40 s^−1^ (n = 5). (G). Fluorescence decay rates are compared with slow current decay rates from constructs I470, I470F, I470C and I470V in 3 mM (black) and 99 mM (grey) K^+^. A slow exponential time constant was mostly obscured by the fast decaying process in I470L, so these are not shown. (H). Inactivation rates from fluorescence in (A–E) in 3 mM and 99 mM K^+^ are calculated using Equation 2, and plotted against each other, and fit by linear regression analysis (slope = 0.30 ± .02, R^2^ = 0.99). The dashed line represents the regression line fit to points in Figure 2F. Current amplitudes shown in (A–E) are normalized to the peaks of both traces, and fluorescence amplitudes in (A–E) are normalized to baseline signal amplitudes at –80 mV.

We next examined the full-length *Shaker* channel to determine if the interaction between I470 and the outer pore is retained even with the long N-terminus and the very fast decay in ion flow that occurs upon depolarization of the membrane potential. Unlike in *Shaker*-IR, where C-type inactivation can be observed directly from current decay, full-length *Shaker* exhibits more complex current decay due to the co-existence of fast N-terminal block and slow pore rearrangement. Therefore, in addition to extracting the rate of slow inactivation from fitting of complex ionic current decay, we used full-length *Shaker* S424C, and performed voltage-clamp fluorimetry in conjunction with two electrode voltage clamp to more easily and accurately track conformational changes in the outer pore that follow C-type inactivation^25^. When current decay and fluorescence traces from *Shaker*-FL S424C were fit to exponential functions, the time constant of fluorescence decay corresponded well with a slower time constant of current decay (Figure 3A).

Next, the residue T449, located just C-terminal to the GYG triplet of the selectivity filter and positioned extracellularly in the Kv1.2 3D structure (see alignment of *Shaker*-related channels in Figure 1), was mutated to valine (T449V) in *Shaker*-FL S424C. Mutation of T449 in *Shaker* antagonizes outer pore rearrangement and, like A463C, is thought to enhance potassium occupancy in the outer pore^20^. When *Shaker*-FL S424C T449V was depolarized, rapid N-type inactivation signal was still observed, but current decay was far less profound and the rapid transient was followed by a much more slowly decaying second phase (Figure 3B), compared to *Shaker*-FL S424C. Fluorescence signals recorded from *Shaker*-FL S424C during depolarization were almost completely abolished in *Shaker*-FL S424C T449V. Taken together, these results show a dramatic reduction of outer pore rearrangement by T449V and show that this alteration in outer pore structure is tracked accurately by S424C fluorescence.

The rates of change in fluorescence were measured in channels containing the same series of conservative mutations at site I470 shown in Figure 2 and were slowed by valine, cysteine and phenylalanine and sped up by leucine (Figure 3C–F). When the same experiments were conducted with an elevated level of extracellular potassium, a slowing of S424C fluorescence kinetics corresponding in time with C-type inactivation was observed for all mutant channels. As with wild type, current decay was fit with multiple exponentials, and compared to the fluorescence traces; time constants from slow current decay rates and fluorescence decay rates corresponded well (Figure 3G). The current decay of *Shaker*-FL I470L was rapid and profound (Figure 3C) and it was not possible to reliably extract rates of current decay that corresponded to a separate slow exponential process. Nevertheless, the data show that the changes in fluorescence, and the underlying conformational changes in the outer pore, are tightly associated with slow inactivation of ionic current.

When S424C fluorescence rates in high extracellular potassium were plotted against those in normal potassium for all constructs tested, regression analysis once again demonstrated linearity (Figure 3H), with a slope showing sensitivity to extracellular potassium across all mutants. Superimposition of the line obtained from the same experiments using the N-terminally deleted channel shows that the slopes are close to identical. The rank order of the rates of inactivation for the *Shaker*-FL mutants is similar, but not identical to the corresponding *Shaker*-IR mutant channels (compare Figure 2F with Figure 3H). Like the *Shaker*-IR mutants, *Shaker*-FL I470C and I470L yield the fastest and slowest rates; however, I470V renders a faster rate than I470F, which is now faster than wild type (I470). Nevertheless, the *Shaker*-FL channels remain sensitive to potassium, favouring our hypothesis that I470 controls coupling in both short and long forms of the *Shaker* channel without greatly impacting potassium sensitivity. Thus, while the N-terminus greatly accelerates the overall rates of C-type inactivation^6^, it does not disrupt coupling of the 470 side-chain with the outer pore.

### Conservative substitutions of I470 modify the interaction of the N-terminus with the inner pore of the full-length Shaker channel

Although elevating extracellular potassium slowed the rates of C-type inactivation of *Shaker*- IR channels and changes in fluorescence of *Shaker*-FL channel to a roughly equal extent, the latter were always faster than the former from the matching *Shaker*-IR mutant regardless of extracellular potassium concentration. The faster rates of full-length *Shaker* are probably due to an effect of the N-terminus as it interacts with the inner pore^6^. The I470 residue is among those that have been proposed to strongly and directly interact with the N-terminus it resides in the mouth of the potassium channel pore^14^.

**Figure 4:**
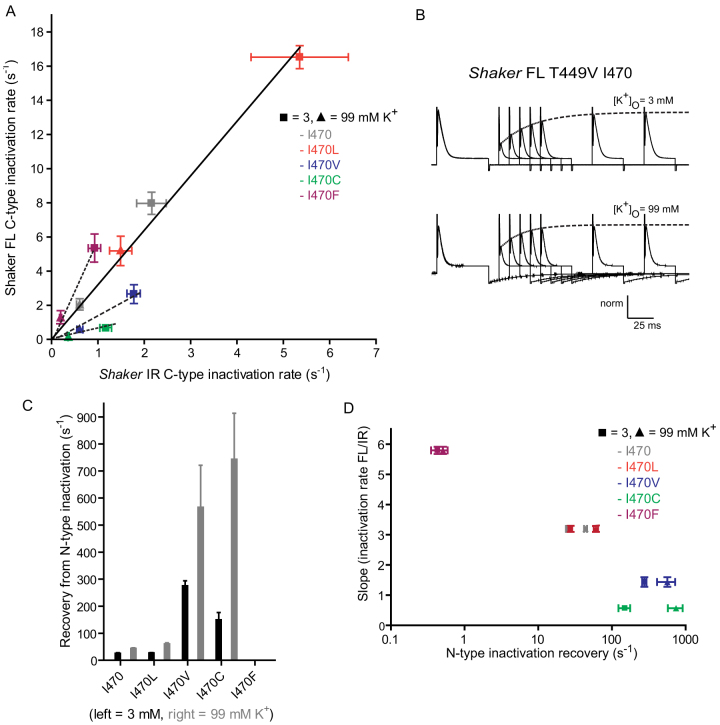
The rate of C-type inactivation of full-length *Shaker* is dictated by the dwell time of the long N-terminus in the inner pore cavity. (A). Current and fluorescence decay rates plotted in Figures 2 and 4 are re-plotted against each other to compare inactivation rates in full length *Shaker* (y-axis) and *Shaker*-IR (x-axis) with the equivalent I470 mutation in the presence of either 3 mM (squares) or 99 mM extracellular potassium (triangles). The black lines represent regression line fits for I470 and the mutant constructs. (B). Ionic currents measured in response to a two pulse protocol used to calculate the off-rates of the N-terminus from its blocking site in 3 mM (top) and 99 mM K+ (bottom). Following a pulse to +60 mV, cells were returned to –100 mV for an increasing period, then re-pulsed to +60 mV. The amplitudes of the second peaks were fit to a single exponential function (dashed lines). (C). Recovery rates from N-type inactivation were calculated from single exponential fits from panel B using Equation 2, and are plotted for the I470 and the four studied mutants. I470F rates were too slow to be visible on the y-axis time scale (n = 6 for I470; n = 5 for I470V, I470C and I470F; n = 4 for I470L). (D). Regression lines are fit to data from each construct from panel A (see inset). The slopes of these curves are then plotted against rates calculated in panel C for each equivalent I470 mutation in the presence of either 3 mM (squares) or 99 mM extracellular potassium (triangles).

To compare the degree of acceleration of outer pore rearrangement by the N-terminus, we re-plotted data from Figures 2 and 3 (Figure 4A) to compare rates with (Y-axis) and without (X-axis) the presence of the N-terminus at normal and high concentrations of extracellular potassium. We noticed that the degree of acceleration induced by the N-terminus was consistent between I470 and I470L (black line), whereas it was greater for I470F (above the black line) and smaller for I470C and I470V (below the black line). While I470F inactivated relatively slowly in the absence of the N-terminus, the intact N-terminus had a stronger effect on inactivation than it did on I470V.

To examine if the effect of the N-terminus was related to the strength of its association with the inner pore, the off-rate of the former was estimated using a two pulse protocol, with the *Shaker* T449V mutant to hinder conformational changes of the selectivity filter; thus, the main contributor to the rapid decay in current after opening is the N-terminus interaction with the inner pore, from which the off-rate can be reasonably estimated.

Brief depolarizing pulses were applied to cells expressing full length T449V channels with the same substitutions at I470. Subsequently, cells were given hyperpolarizing pulses of increasing duration, followed by a second depolarizing pulse to measure the peak amplitude (Figure 4B). With increasing inter-pulse durations, the peak amplitudes during the second pulse recovered to the levels of those of the first pulse. The amplitudes of the second peaks for all constructs could all be fit to single exponentials, suggesting that the recovery from block was rate-limited by a single gating transition that reflected the unbinding of the N-terminus; thus, these rates are inversely proportional to the affinity of the peptide for the inner cavity (Figure 4C).

We also calculated the slopes of regression lines between full length and truncated constructs shown in Figure 4A for the remaining mutants falling above or below the solid line (Figure 4A). We noticed that the stronger the affinity of the peptide for the inner cavity correlated with a comparatively stronger accelerating effect of the N-terminus on the rate of fluorescence decay and reflected C-type inactivation (Figure 4D). Specifically, the binding of the N-terminus to I470F was also stronger than for I470V; we suspect that this speeds the rate of slow inactivation to a greater extent in the former, leading to the altered rank order of inactivation rates of the mutant *Shaker*-FL channels in Figure 3H.

## Discussion

Conservative mutations of *Shaker* I470 modify the rate of C-type inactivation without affecting its sensitivity to extracellular potassium when its concentration is elevated. The retention of wild type potassium sensitivity amongst all I470 mutants tested suggests that altered inactivation rates of the mutants are likely not secondary to altered coordination of this cation by the outer pore at these concentrations. Together with previous studies^13^,^15^, our findings support a model in which I470 constitutes an initial part of an allosteric pathway that undergoes a re-orientation as the inner gate opens and widens, impinging upon the outer pore to change its conformation and limit conduction, as has been suggested for F103 in the *KcsA* channel based on functional and structural information. Our data are consistent between the full-length and inactivation-removed *Shaker* channels; thus, the allosteric signal emanating from the inner pore and I470 is also important for conformational rearrangement of the outer pore in the former.

The effects of I470 mutants contrast with that of A463C, also near the inner activation gate, which slowed the rate of inactivation in a manner that was insensitive to changes in concentration of extracellular potassium. Insensitivity of A463C to extracellular potassium is consistent with previous findings suggesting that its slowed rate of C-type inactivation is due to permanent occupancy of the outer pore by potassium and not to an effect of the mutation on the conformational change leading to inactivation^21^,^22^. The mutation T449V also slows inactivation and removes sensitivity to potassium and, when combined with the mutation I470C, almost completely inhibits slow inactivation^16^,^17^. Although mutations of I470 may have a more subtle influence on the binding affinity of potassium, they do not alter the maximum effect of this cation, unlike mutations at A463 and T449, supporting a role for the former residue in slow inactivation that does not directly impact potassium occupancy.

Unlike the *KcsA* channel, the *Shaker* channel also has a long N-terminus that is responsible for fast inactivation and speeds slow inactivation. A comparison of rates of C-type inactivation between full-length *Shaker* and *Shaker*-IR suggests that the N-terminus, as well as inhibiting potassium entry into the pore, interacts with the inner pore to separately promote conformational changes in the selectivity filter^6^. These data suggest that the effect of the N-terminus on slow inactivation is correlated with the strength of its association with the inner pore, which, in turn, is influenced by I470. Thus, this residue plays a dual role in the control of slow inactivation in Kv channels that possess a long N-terminus and fast inactivation.

Lastly, in contrast to the *KcsA* channel, *Shaker*-related channels possess a voltage-sensing domain that could interact with the outer pore to impact slow inactivation; this is supported by studies showing that changes in voltage sensor and pore conformation by tracking changes in fluorescence correlate in time with C-type inactivation^25^. However, a more recent analysis suggests that voltage-sensor movement is faster than C-type inactivation in *Shaker* and can be modified independently from the pore; furthermore, a protein from *Ciona intestinalis* that contains a potassium-like voltage sensing domain but lacks a pore undergoes conformational changes during depolarization that are similar to those of the *Shaker* voltage sensor^26^. Together, the data suggest that the outer pore and voltage sensing elements of *Shaker* do not directly interact and influence each other during prolonged depolarization.

## Methods

### DNA and RNA preparation

A full length *Shaker* clone in pGW1 was a kind gift from Dr. Richard Horn (Thomas Jefferson University, Philadelphia, PA). *Shaker* was excised from pGW1 by PCR amplification from the 5′ and 3′ ends using Phusion polymerase (Fisher Scientific, Nepean, ON) while simultaneously engineering recognition sites for *Hind*III (5′) and *EcoR*I (3′) restriction enzymes on either end of the open reading frame. The resulting linear DNA fragment was then digested with those enzymes, and ligated into a similarly digested pBluescript II SK+ vector (Fermentas, Burlington, ON) using T4 ligase (NEB). Single point mutations were generated using the Quikchange kit (Stratagene, La Jolla, CA) as recommended by the manufacturer. By this method, site S424 was mutated to cysteine, to report upon C-type inactivation^25^. All channel constructs used in this study were made in the background of S424C. All DNA constructs were linearized past the 3′ end of the coding sequence with *Sac*II, following which RNA was transcribed from the T7 promoter using the T7 mMessage mMachine Kit (Life Technologies, Carlsbad, CA), as described by the manufacturer.

### Xenopus oocyte preparation, two-microelectrode voltage clamp and voltage clamp fluorimetry

For two electrode clamp and voltage clamp fluorimetry, *Xenopus* oocytes were prepared and maintained for recordings as described^27^. Recordings were performed in ND96-type solutions, which contained, in mM: NaCl, 96; KCl, 3; HEPES, 10; MgCl_2_, 1; CaCl_2_, 2; adjusted to pH 7.4 with NaOH. Modified versions of this solution containing varying concentrations of extracellular K^+^ were made by replacing NaCl with KCl up to the desired concentration of K^+^ ions, up to a maximum K^+^ concentration of 99 mM. Recordings were performed using tetramethylrhodamine maleimide (Life Technologies), as described previously^27^. Each fluorescence trace was subtracted from a paired recording from the same oocyte, where no depolarizing pulse was given, to control for any bleaching of the fluorophore. Data shown using multiple concentrations of K^+^ are matched from the same oocytes.

### Data analysis

Data were analyzed using pClamp10 (Axon Labs), Origin 8 (OriginLabs), Microsoft Excel (Microsoft) and Graphpad Prism 4.0 (Graphpad Software Inc). All data are presented as means ± S.E.M, and “n” refers to the number of oocytes tested. All comparisons employed one-way ANOVA to prevent error from multiple comparisons. Significance was assessed using the *post-hoc* Bonferroni test. P < 0.05 was used as a threshold of significance. Current and fluorescence decay, and peak current recovery from N-terminal block time courses were fit to single exponentials based on an equation of the form: 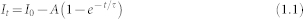
where *I_t_* and *I_0_* represent current amplitudes at times t and 0 seconds, respectively. In the case of *Shaker*-IR I470V, a double exponential function was required for fitting, of the form: 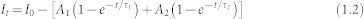
Inactivation rates were calculated from time constants based on the relationship: 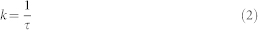
and are presented in units of s^−1^.

## Author Contributions

C.P. carried out the experiments, analyzed the data and made the figures for both the initial submission and revision. The experiments were conceived and designed by C.P., D.F. and E.A. who together also provide an interpretation of the data. The manuscript was edited, both initially and upon revision, by C.P., D.F. and E.A. Finally, the study materials and approaches were funded by D.F. and E.A. whereas C.P. carried funding for his own stipend. Eric Accili is the recipient of a Canada Research Chair (Tier 2).

## Supplementary Material

Supplementary Information Supplementary Information
