# Supplementary material for: Allosteric coupling of the inner activation gate to the outer pore of a potassium channel
Source: Sci Rep. 2013 Oct 23;3:3025. doi: 10.1038/srep03025 (PMC3806241; doi:10.1038/srep03025)
Supplement: Supplementary Information [file srep03025-s1.pdf]

Allosteric coupling of the inner activation gate to the outer pore of a potassium channel

Christian J. Peters[1], David Fedida[2], Eric A Accili[1]*

[1]Department of Cellular and Physiological Sciences, and [2]Department of Anesthesiology, Pharmacology, and Therapeutics, University of British Columbia, Vancouver, British Columbia, V6T 1Z3

Supplementary Information

Supplementary Figure 1. Extracellular potassium does not slow current decay of *Shaker* IR A463C.

A. Normalized current traces from *Shaker* IR A463C in response to a voltage pulse to +60 mV from –80 mV, in solutions containing 3 mM (black) and 99 mM (grey) extracellular K^+.

B. Rates of inactivation, determined from current traces as shown in ‘A’ using Equation 1, are plotted against a range of potassium concentrations for *Shaker* IR (see Figure 2A) and *Shaker* IR A463C.

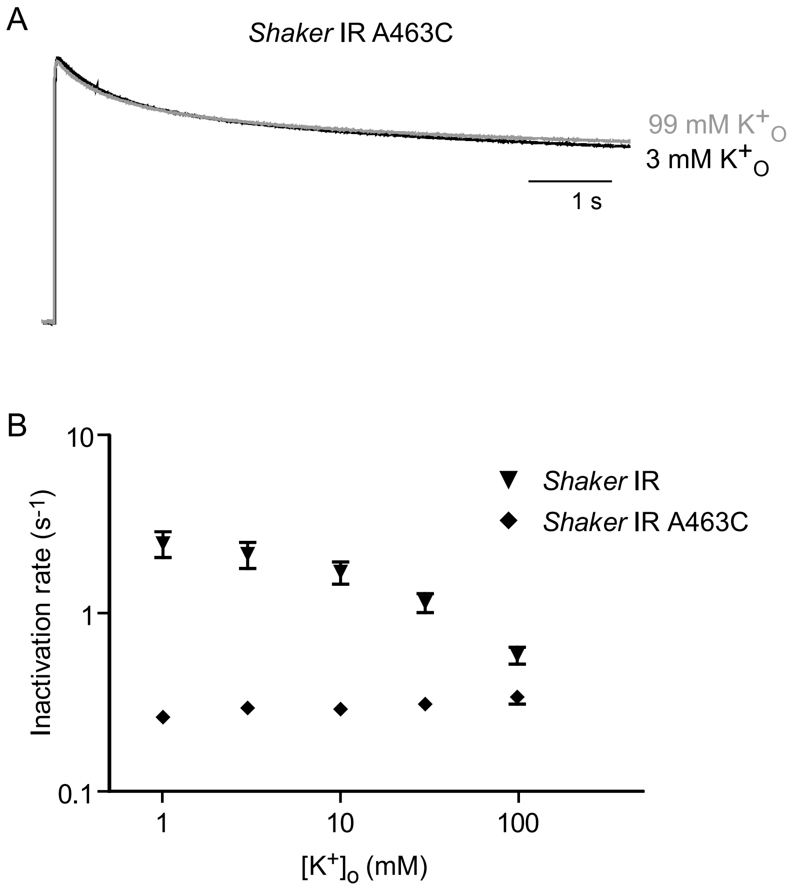
A
Shaker IR A463C
99 mM K+O
3 mM K+O
1 s
B
10
1
0.1
Inactivation rate (s-1)
Shaker IR
Shaker IR A463C
1
10
100
[K+]o (mM)
